# New Deep Learning Genomic-Based Prediction Model for Multiple Traits with Binary, Ordinal, and Continuous Phenotypes

**DOI:** 10.1534/g3.119.300585

**Published:** 2019-03-11

**Authors:** Osval A. Montesinos-López, Javier Martín-Vallejo, José Crossa, Daniel Gianola, Carlos M. Hernández-Suárez, Abelardo Montesinos-López, Philomin Juliana, Ravi Singh

**Affiliations:** * Facultad de Telemática; ** Facultad de Ciencias, Universidad de Colima, Colima, 28040, México; † Departamento de Estadística, Universidad de Salamanca, c/Espejo 2, Salamanca, 37007, España; ‡ International Maize and Wheat Improvement Center (CIMMYT), Apdo. Postal 6-641, 06600, Ciudad de México, México; § Departments of Animal Sciences, Dairy Science, and Biostatistics and Medical Informatics, University of Wisconsin-Madison, Wisconsin 53706; †† Departamento de Matemáticas, Centro Universitario de Ciencias Exactas e Ingenierías (CUCEI), Universidad de Guadalajara, 44430, Jalisco, México

**Keywords:** deep learning, multiple-trait, mixed phenotypes (binary ordinal and continuous), genomic selection, plant breeding, Genomic Prediction, GenPred, Shared Data Resources

## Abstract

Multiple-trait experiments with mixed phenotypes (binary, ordinal and continuous) are not rare in animal and plant breeding programs. However, there is a lack of statistical models that can exploit the correlation between traits with mixed phenotypes in order to improve prediction accuracy in the context of genomic selection (GS). For this reason, when breeders have mixed phenotypes, they usually analyze them using univariate models, and thus are not able to exploit the correlation between traits, which many times helps improve prediction accuracy. In this paper we propose applying deep learning for analyzing multiple traits with mixed phenotype data in terms of prediction accuracy. The prediction performance of multiple-trait deep learning with mixed phenotypes (MTDLMP) models was compared to the performance of univariate deep learning (UDL) models. Both models were evaluated using predictors with and without the genotype × environment (G×E) interaction term (I and WI, respectively). The metric used for evaluating prediction accuracy was Pearson’s correlation for continuous traits and the percentage of cases correctly classified (PCCC) for binary and ordinal traits. We found that a modest gain in prediction accuracy was obtained only in the continuous trait under the MTDLMP model compared to the UDL model, whereas for the other traits (1 binary and 2 ordinal) we did not find any difference between the two models. In both models we observed that the prediction performance was better for WI than for I. The MTDLMP model is a good alternative for performing simultaneous predictions of mixed phenotypes (binary, ordinal and continuous) in the context of GS.

Many times a breeder wishes to genetically improve more than one trait but with mixed phenotypes. For example, grain yield (GY) measured on a continuous scale, presence or absence of a certain disease or level of infection of a certain disease (non-infected, low level of infection, middle level of infection, high level of infection and totally infected). However, when breeders wish to analyze the data with mixed phenotypes, (a) they usually analyze the data using univariate statistical models; (b) they subject the discrete variables (binary and ordinal) to some numerical score (using transformations), so that all variables can be treated as continuous, and then they apply conventional multivariate analysis; and (c) the continuous variables are discretized through some grouping criteria, which allows all variables to be treated as discrete and then analyzed under a multivariate model for discrete responses. However, these three approaches involve some level of subjectivity since the first approach does not take into account the correlation between traits, the second method introduces a high level of subjectivity in the numerical scoring scheme and the third solution produces considerable loss of information due to the discretization process of the continuous variables (Krzanowski 1983). Although there are multivariate techniques for regression analysis for association studies and prediction modeling (Montesinos-López *et al.*, 2016), versatile regression models for mixed outcomes are lacking, since conventional multivariate statistical tools generally rely on the assumption that the data, or suitable transformations of them, follow a normal distribution.

Plant breeders have a long tradition using multivariate models for association studies and prediction modeling; however, with the new paradigm called genomic selection (GS) proposed by Meuwissen *et al.* (2001), there is renewed interest in multivariate modeling to exploit the correlation between traits to improve parameter estimates or prediction accuracy. Jia and Jannink (2012) provided evidence that multivariate analyses outperform univariate analysis when there is at least moderate correlation between traits. Jiang *et al.* (2015) and Montesinos-López *et al.* (2016) came to the same conclusion in favor of multivariate analysis. He *et al.* (2016) and Schulthess *et al.* (2017) also found that, compared to single-trait analysis, multivariate analysis could improve prediction accuracy for correlated traits. However, the application of traditional multivariate models in the context of GS is not straightforward due to the problem of the large amount of independent variables (marker information and environmental information) and few observations (lines), a problem commonly known as “large p and small n”. For this reason, in recent years, intensive research has been conducted to develop statistical models (or adapt conventional models) for the context of genome-wide association analysis and genome-enabled prediction (GP). In the context of univariate and multivariate models with continuous phenotypes for GP, Bayesian models have proved to be more efficient than models based on maximum likelihood or restricted maximum likelihood because they are better suited for dealing with data sets with large p and small n. As evidence of this, the term “Bayesian alphabet” was coined; it refers to the growing number of letters of the alphabet used to denote various Bayesian linear regressions used in GP that differ in the priors adopted, while sharing the same sampling model (Gianola 2013). Gianola (2013) also pointed out that the Bayesian alphabet is of paramount importance in whole-genome prediction of phenotypes, but has somewhat doubtful inferential value, at least when the sample size is such that n ≪ p.

However, to date there are no Bayesian regression models for GP for mixed phenotypes due to the difficulty of developing efficient analytic Gibbs samplers to perform a Markov Chain Monte Carlo (MCMC) algorithm for approximating a specific multivariate probability distribution, when direct sampling is difficult. But nowadays there are computationally efficient methodologies for predicting multiple-trait (called multiple-output) response variables (phenotypes) in deep learning (DL), which is a part of a broader family of machine learning methods based on learning data representations, as opposed to task-specific algorithms. In general terms, machine learning is devoted to developing and using algorithms that learn from raw data in order to make predictions (Wiley 2016).

Inspiration for DL models is rooted in the functioning of biological nervous systems. These models are not new because their roots trace back to the introduction of the McCulloch-Pitts (MCP) model, which is considered the ancestor of the artificial neural model (McCulloch and Pitts 1943) that has now gone mainstream thanks to its practical applications and availability in terms of consumable technology and affordable hardware. An artificial neural network (ANN) models the relationship between a set of input signals and an output signal using a model derived from our understanding of how a biological brain responds to stimuli from sensory inputs. Similar to how the brain uses a network of neurons (interconnected cells or units) to create a massive parallel processor, ANN uses a network of artificial neurons to solve learning problems (Lantz 2015). For this reason, an ANN is described as a directed graph whose nodes correspond to neurons and whose edges correspond to links between them. Each neuron receives as input a weighted sum of the outputs of the neurons connected to its incoming edges (Figure 1). Feedforward networks (Figure 1) are those in which the underlying graph does not contain cycles (Shalev-Shwartz and Ben-David 2014). Thanks to major innovations in the field of neural networks, a technique that is known as deep learning has emerged. The term deep refers to the fact that we can now train different ANN configurations with more than a single hidden layer, such as the conventional multilayer perceptron, which has shown to have better generalization capabilities (Goodfellow *et al.*, 2016). The adjective “deep” applies not in itself to the knowledge acquired, but to the way in which knowledge is acquired (Lewis 2016). In other words, DL is a subfield of machine learning that generalizes conventional neural networks to work with more than two hidden layers and more neurons; it is devoted to building prediction algorithms that explain and learn a high and low level of abstraction (Gibson and Patterson 2017).

**Figure 1:**
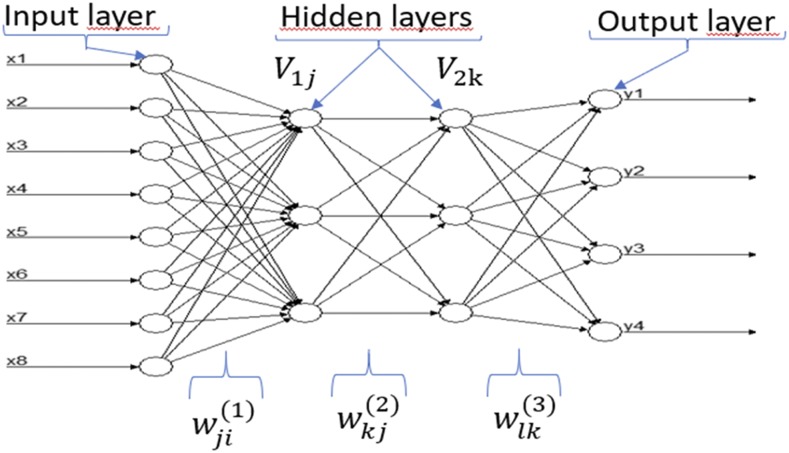
Example of a feedforward neural network with 8 input variables (x1,…,x8), four output variables (y1, y2, y3, y4), and two hidden layers with three neurons each.

For this reason, DL models have been implemented in many areas of knowledge: prediction of time series (Dingli and Fournier 2017); language processing (Goldberg 2016); self-driving cars (Liu *et al.*, 2017); predicting breast, brain (Cole *et al.*, 2017) or skin cancer using personalized medicine based on Biobank-data, voice search and voice-activated intelligent assistants (LeCun *et al.*, 2015); automatically adding sound to silent movies; automatic translation of text and images (LeCun *et al.*, 2015); automatic text generation; automatic handwriting generation (LeCun *et al.*, 2015); image recognition (LeCun *et al.*, 2015); automatic image captioning (that is, given an image, the system must generate a caption that describes the content of the image); automatic colorization; advertising; predicting earthquakes (Rouet-Leduc *et al.*, 2017); energy market price forecasting (Weron 2014); and genomic prediction (Montesinos-López *et al.*, 2018a, b).

There are also many applications of DL and machine learning for the biological sciences. For example, Fox *et al.* (2018) applied DL for predicting blood glucose trajectories, Menden *et al.* (2013) predicted cancer cell sensitivity to drugs with machine learning, Alipanahi *et al.* (2015) used DL for predicting the sequence specificities of DNA- and RNA-binding proteins, and Tavanaei *et al.* (2017) applied DL for predicting tumor suppressor genes and oncogenes. Recently, Montesinos-López *et al.* (2018a, b) have shown how to apply DL methods with densely connected network architecture and several hyperparameter combinations to extensive plant breeding data involving different traits and environments. Compared with the standard genomic model and ignoring genotype × environment interaction (G×E), the DL method was better than the conventional genomic models in terms of prediction accuracy. The previously mentioned authors also developed DL models and methods for multiple traits (all continuous traits) and compared their prediction accuracy with a Bayesian multiple-trait multiple-environment model. Among models without genotype × environment interaction, the multiple-trait DL model was the best, while among models with genotype × environment interaction, the Bayesian multiple-trait multiple-environment model was superior. However, no models or methods have been developed for genomic selection and prediction in animal and plant breeding that can incorporate together binary, ordinal and continuous traits and their G×E when collected in different environments.

For the reasons outlined above, we applied DL methods for predicting multiple traits with mixed phenotypes (binary, ordinal and continuous traits). We also compared their performance with that of univariate deep learning models where a model was individually trained for each trait. To evaluate the performance of both models (multiple-trait and univariate-trait deep learning), we used seven wheat data sets from the Global Wheat Breeding Program of the International Maize and Wheat Improvement Center (CIMMYT). The performance of both models in terms of prediction accuracy was evaluated using cross-validation in terms of average Pearson’s correlation (APC) for continuous traits and in terms of the percentage of cases correctly classified (PCCC) for binary and ordinal traits.

## Materials and Methods

### Multiple-trait deep learning with mixed phenotypes (MTDLMP) model

While there are different DL architectures (densely connected networks, convolutional networks, recurrent networks, etc.), in this paper we focus on a standard densely connected network. Details of each type of network, its assumptions and input characteristics can be found in Gulli and Sujit (2017), Angermueller *et al.* (2016) and Chollet and Allaire (2017). A densely connected network does not assume a specific structure in the input features. In general, the basic structure of a densely connected network consists of an input layer, L output layers (for multiple-trait modeling) and multiple hidden layers between the input and output layers. This type of neural network is also known as a feedforward neural network (see Figure 1).

In this paper we implemented the architecture shown in Figure 1 for the seven data sets used in this study with 1, 2 and 3 hidden layers and number of neurons (from 10 to 500 with increases of 20). As input variables ($X=\{x_{ip}\},$ i = 1,2,..,n; *P* = 1,2,..,$\;N_{1}$) for the proposed deep learning model, we included predictor variables as illustrated in Figure 1, since we used as input information for each line the resulting covariates of concatenating information on environments, information on markers through the Cholesky decomposition of the genomic relationship matrix and information on the genotype × environment (G×E) interaction. This meant that first we built the design matrices of environments ($Z_{E})$, genotypes ($Z_{G})$ and  G×E ($Z_{GE});$ then we obtained the Cholesky decomposition of the genomic relationship matrix (G). Then we post-multiplied the design matrix of genotypes by the transpose of the upper triangular factor of the Cholesky decomposition ($Q^{T}$), $Z_{G}^{*}=Z_{G}Q^{T}$, and finally the G×E term was obtained as the product of the design matrix of the G×E term post-multiplied by the Kronecker product of the identity matrix of order equal to the number of environments and $Q^{T}$, that is, $\;Z_{GE}^{*}=Z_{GE}(I_{I}⊗Q^{T}$). Finally, the matrix with input covariates used for implementing both deep learning models was equal to $X=\left[Z_{E},Z_{G}^{*},\;Z_{GE}^{*}\right]$. The input variables ($x_{ip}$) are connected to the neurons in the first hidden layer via weights. The input variables simply pass the information to the units in the first hidden layer. The net input into the jth hidden unit in the first hidden layer is $h_{1j}=\overset{N_{1}}{\underset{p=1}{\sum }}w_{jp}^{\left(1\right)}x_{p}+b_{j}^{\left(1\right)}$, where $N_{1}$ is the total number of input variables, $w_{jp}^{\left(1\right)}$ is the weight of input unit p to hidden unit j in the first hidden layer, $x_{p}$ is the value of the pth input variable and $b_{j}^{\left(1\right)}$ is a bias specific to neuron (unit) j in layer 1. Then the jth hidden unit in the first hidden layer applies an activation function to its net input and outputs $V_{1j}=g_{1}(h_{1j})$ for $j=1,\ldots ,\;N_{2}$. Similarly, neuron k in the second hidden layer receives a net input $h_{2k}=\overset{N_{2}}{\underset{j=1}{\sum }}w_{kj}^{\left(2\right)}V_{1j}+b_{k}^{\left(2\right)}$, where $N_{2}$ is the total number of input neurons that come from hidden layer 1 to neuron k, $w_{kj}^{\left(2\right)}$ is the weight from unit j of layer 1 that goes to unit k in hidden layer 2, $V_{1j}$ is the value of the output of unit j in layer 1 and $b_{k}^{\left(2\right)}$ is a bias specific term to neuron k in layer 2. Then the kth hidden unit in the second hidden layer applies an activation function to its net input and outputs $V_{2k}=g_{2}(h_{2k})$ for k=1,…, M. Similarly, output unit t with t=1,2,..,L, receives a net input of $h_{3t}=\overset{M}{\underset{k=1}{\sum }}w_{tk}^{\left(3\right)}V_{2k}+b_{t}^{\left(3\right)}$, where M is the number of hidden units from hidden layer 2, $w_{tk}^{\left(3\right)}$ represents the weight from hidden unit k in layer 2 to output t. Finally, the prediction of an individual in trait t is obtained as: ${\hat{y}}_{t}=g_{3}(h_{3t})$. It is important to point out that in the output layer ($g_{3})$, the sigmoid, softmax and rectified linear units (ReLU) activation functions were used for binary, ordinal and continuous traits, respectively.

Successful implementation of most DL models requires an appropriate hyperparameter tuning process. However, implementing a feedforward neural network is challenging because it requires a tuning process of the following hyperparameters: number of units (U), number of layers, number of epochs (E), type of regularization method and type of activation function. Based on the literature review, we decided to use the ReLU, sigmoid and softmax activation functions for the continuous, binary and ordinal response (output) variables, respectively, while for the hidden layers, we used the ReLU activation function. As for the type of regularization, we chose dropout regularization for training the models (Gulli and Sujit 2017; Chollet and Allaire 2017; Srivastava *et al.*, 2014), and for the hidden layers we used 1, 2 and 3 hidden layers.

Concerning the number of epochs and number of units in the hidden layers, we performed a grid search. The grid search was done with number of epochs from 1 to 100 and number of units between 10 to 500 with increases of 20. For more details on model selection in DL models, we suggest reading the papers of Montesinos-López *et al.* (2018a, b), where the authors evaluate the prediction performance of univariate and multivariate DL models for continuous response variables. It is important to point out that we also implemented the univariate counterpart of the MTDLMP model described at the beginning of this section, where each trait was implemented using a univariate deep learning (UDL) model with exactly the same architecture given in Figure 1, except that it only had one output variable. When the output variable was continuous, ReLU activation was used, whereas the sigmoid and softmax activation functions were used for the binary and ordinal response (output) variables, respectively. Both the MTDLMP and UDL models were implemented in the keras package (Chollet and Allaire 2017) in the open-source software R (R Core Team 2018).

### Evaluating prediction performance with cross-validation

The prediction accuracy of both models (MTDLMP and UDL) was evaluated with an outer CV while an inner CV was used for tuning the hyperparameters. The outer CV consisted of a fivefold CV, where the original data sets were partitioned into five subsamples of equal size and each time four of them were used for training (TRN) and the remaining one for testing (TST). In our outer CV, one observation cannot appear in more than one fold. In the design, some lines can be evaluated in some, but not all, target environments, which mimics a prediction problem faced by breeders in incomplete field trials. For this reason, our cross-validation strategy is exactly the same as the strategy denoted as CV2 that was proposed and implemented by Jarquín *et al.* (2017), where a certain portion of test lines (genotypes) in a certain portion of test environments are predicted since some test lines that were evaluated in some test environments are assumed to be missing in others.

The metric used to measure the prediction accuracy of both models was Pearson’s correlation for continuous traits and the percentage of cases correctly classified (PCCC) for the binary and ordinal variables. They were calculated from each trait-environment combination for each of the testing sets and the average of all folds was reported as a measure of prediction performance. It is important to point out that, to avoid biased results, the tuning step was done in each fold using only the training set.

For the tuning process we implemented the inner CV with the proposed grid for the number of epochs and units, and 20% of each training set was used as a validation set (validation-inner). Due to the amount of data and the complexity of the MTDLMP and UDL models, the training process requires a lot of time for the tuning process; for this reason, the training was performed using the internal capabilities of keras, where we set the validation_split argument on the fit() function to 20% of the size of each of our training data sets. This automatic validation procedure of keras implemented the inner CV and evaluated the performance of the model on the validation data set for each epoch and avoided implementing manual k-fold cross-validation for the inner CV, which requires more computational resources (Chollet and Allaire 2017).

#### Experimental data sets:

In this study we used the data set used by Juliana *et al.* (2018). The data used belong to four elite yield trial (EYT) nurseries from the Global Wheat Program of the International Maize and Wheat Improvement Center (CIMMYT). The EYT nurseries were planted in mid-November because that is the best time to plant CIMMYT’s yield trials. Bed and flat planting systems in optimally irrigated environments received 500 mm of water at the Norman E. Borlaug Research Station, Ciudad Obregon, Sonora, Mexico. The nurseries were sown in 39 trials, each comprising 28 lines and two high-yielding checks (Kachu and Borlaug) that were arranged in an alpha lattice design with three replications and six blocks. The nurseries were evaluated for the following traits: number of days from germination to 50% spike emergence (days to heading, DTHD), number of days from germination to 50% physiological maturity (days to maturity, DTMT), grain yield (GY, tons per hectare) and plant height (Height, centimeters). All these nurseries were evaluated during four seasons 2013-2014 (EYT 13-14; here called **data set 1**), 2014-2015 (EYT 14-15; called **data set 2**), 2015-2016 (EYT 15-16; called **data set 3)** and 2016-2017 (EYT 16-17; called **data set 4**).

Data set 1 included 767 lines, data set 2 had 775 lines, data set 3 comprised 964, and data set 4 had 980 lines (Juliana *et al.*, 2018). In addition, in each season we studied six environments resulting from the level of irrigation (IR) and planting system (bed or flat) which we called: Bed2IR, Bed5IR, Flat5IR, FlatDrip, EHT and LHT. However, all these environments were not evaluated in all seasons (data sets). In **data set 1**, only environments Bed5IR, EHT, Flat5IR and LHT were evaluated. In **data set 2**, the evaluated environments were: Bed2IR, Bed5IR, EHT, Flat5IR and LHT. In **data set 3**, the evaluated environments were: Bed2IR, Bed5IR, Flat5IR and FlatDrip, where 5IR and 2IR refer to 5 and 2 irrigation levels, EHT denotes early heat, LHT is late heat, and bed and flat are two different planting systems. In **data set 4**, the evaluated environments were: Bed5IR, EHT, Flat5IR and FlatDrip.

It is important to point out that here we used the BLUEs of each of the lines obtained (as suggested by Juliana *et al.*, 2018) adjusted for trials, blocks and replications in each data set. Three of the four traits were discretized because the original data sets are continuous, only to illustrate the MTDLMP model. Traits DTHD and DTMT were discretized at quantiles 33.33% and 66.67% (in **data sets 1** and **2**) to obtain three categories, while trait Height was discretized at quantile 50% to obtain 2 categories (in **data sets 1, 2, 3** and **4**) the discretization process was done in each environment of each data set. For **data sets 3** and **4**, traits DTHD and DTMT were discretized at quantiles 20%, 45%, 70% and 90%.

**Data set 5** is part of **data set 3**; for this reason, the phenotypic information and genomic information were obtained in the same way as in **data set 3**; however, only 964 lines had complete data of the total 980 lines under study in **data set 3.** But now the traits measured in **data set 5** were grain color (GC) (1 = yes, 2 = no), leaf rust (ordinal scale with 5 points), stripe rust (ordinal scale with 3 points) and GY, which is a continuous trait. **Data set 6** and **data set 7** are part of the wheat yield trial (YT) nurseries from CIMMYT’s Global Wheat Breeding Program. For **data set 6,** the number of lines used was 945, and for **data set 7**, 1145 wheat lines were used. A continuous trait (grain yield, GY) and an ordinal trait (lodging; ordinal scale of 5 points) were measured on both data sets.

##### Genotypic data:

All 4,368 lines evaluated in the four seasons (nurseries) comprising the EYT of **data sets 1**, **2**, **3**, and **4** were genotyped using genotyping-by-sequencing (GBS) (Elshire *et al.*, 2011; Poland *et al.*, 2012) at Kansas State University, using an Illumina HiSeq2500 for obtaining genome-wide markers. Marker polymorphisms were called across all lines using the TASSEL (Trait Analysis by Association Evolution and Linkage) GBS pipeline (Glaubitz *et al.*, 2014) and anchored to the International Wheat Genome Sequencing Consortium’s (IWGSC) first version of the reference sequence (RefSeq v1.0) assembly of the bread wheat variety Chinese Spring. Markers with more than 60% missing data, less than 5% minor allele frequency and percent heterozygosity greater than 10% were removed and we obtained 2,038 markers. Missing marker data were imputed using LinkImpute (Money *et al.*, 2015) implemented in TASSEL (Bradbury *et al.*, 2007), version 5. The lines were also filtered for more than 50% missing data, found in 3,485 lines (767 lines from **data set 1**, 775 lines from **data set 2**, 964 lines from **data set 3** and 980 lines from **data set 4**) (Juliana *et al.*, 2018). The lines used in **data sets 5**, **6**, and **7** were genotyped with the same marker system that was used for the other data sets.

### Data availability

All seven data sets (Data Sets 1-7) including phenotypic and genotypic data plus the Supplementary Material with the 14 figures (Figure D1_SA, D1_SB-D7_Sa, D7_SB) can be downloaded from the following link: http://hdl.handle.net/11529/10548140. 

## Results

The results are given in seven main sections. Each section describes the results of one data set. Each section is divided into two subsections, one that includes a descriptive analysis and another that reports the genomic-enabled prediction accuracy of the proposed models.

### Data set 1

**Figure 2:**
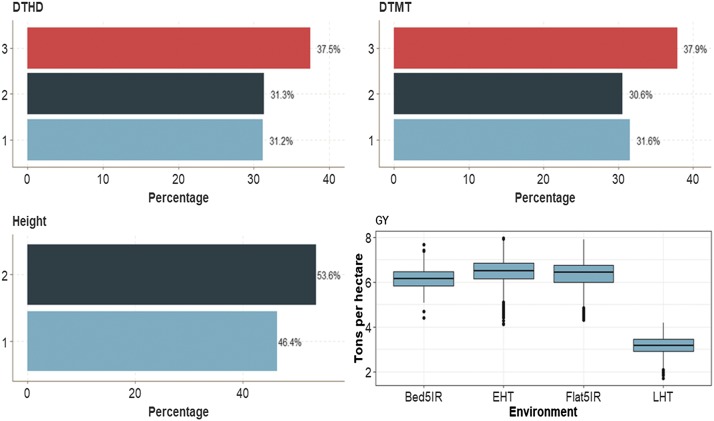
Percentage of each ordinal response for *data set 1* across environments for traits days to heading (DTHD), days to maturity (DTMT) and Height. Boxplot of trait grain yield (GY) for each environment.

This data set had four traits (one binary, two ordinal and one continuous). Across environments, the binary trait (Height) had 53.6% of cases for category 2 and 46.4% for category 1 (Figure 2). Traits DTHD and DTMT had similar distributions of individuals between categories. The average GY was around 6 ton/ha for three environments (Bed5IR, EHT and Flat5IR) and around 3 ton/ha in environment LHT (Figure 2).

**Figure 3:**
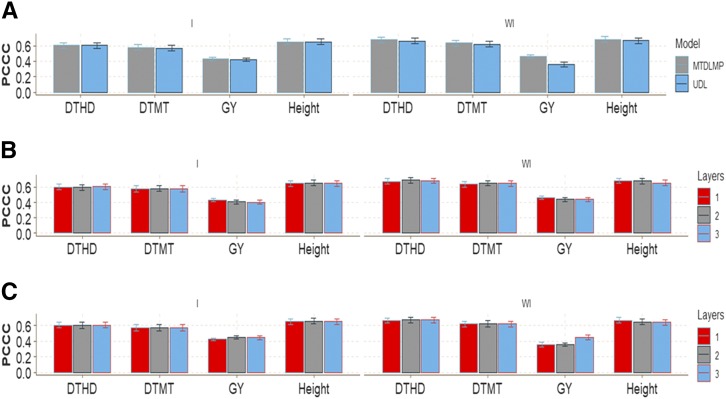
Prediction accuracy of *data set 1* in terms of percentage of cases correctly classified (PCCC) for traits days to heading (DTHD), days to maturity (DTMT) and Height and in terms of average Pearson’s correlation for trait grain yield (GY). (**A**) Prediction accuracy of MTDLMP and UDL models with the G×E term (I) and without the G×E term (WI) for each trait with 1 layer; (**B**) prediction accuracy with different numbers of layers (1, 2 and 3) across environments with the MTDLMP model with the G×E term (I) and without the G×E term (WI); and (**C**) prediction accuracy obtained with different numbers of layers (1, 2 and 3) with the UDL model with the G×E term (I) and without the G×E term (WI).

There were no major prediction accuracy differences in this wheat data set between the MTDLMP and UDL models using 1 layer when G×E interaction was taken into account (I) in any of the traits under study (Figure 3a). However, when G×E interaction was ignored (WI), we found statistical differences in trait GY, with better performance under the MTDLMP model (Figure 3a), and in average Pearson’s correlation (APC), the MTDLMP was superior to the UDL in prediction accuracy by 22.44%. With two layers, we only found statistical differences between the MTDLMP and UDL models in trait GY, where the MTDLMP was again superior (Figure D1_SA; Supplementary material, hdl:11529/10548140); however, with three layers, we did not find any statistical differences between the two models (Figure D1_SB; Supplementary material, hdl:11529/10548140). The PCCC using 1 layer for the binary and ordinal traits (DTHD, DTMT and Height) ranged from 0.5697 to 0.6815, while the APC for the GY trait ranged from 0.3593 to 0.4633 (Figure 3a). Also, when comparing the prediction accuracy using different numbers of layers (1, 2, and 3) under the MTDLMP model (Figure 3b) and under the UDL model (Figure 3c), we did not find statistical differences in terms of prediction performance between using 1, 2, or 3 layers with the G×E interaction term (I) and only found statistical differences between using 1, 2 and 3 layers in trait GY without the G×E interaction term (WI).

### Data set 2

**Figure 4:**
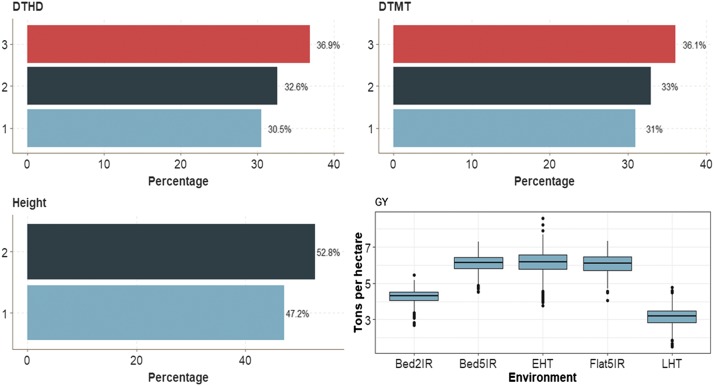
Percentage of each ordinal response for data set 2 across environment for traits days to heading (DTHD), days to maturity (DTMT) and Height. Boxplot of trait grain yield (GY) for each environment.

Trait Height (binary trait) across environments had 52.8% of cases in category 2 and 47.2% in category 1. Traits DTHD and DTMT had similar distribution of individuals in each of the 3 categories. In trait GY, the average GY was 6 ton/ha for three environments (Bed5IR, EHT and Flat5IR), around 3 ton/ha in environment LHT and around 4.3 tons/ha in environment Bed2IR (Figure 4).

**Figure 5:**
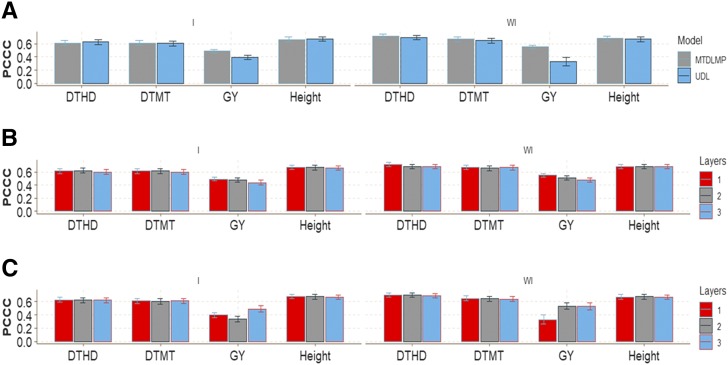
Prediction accuracy of *data set 2* in terms of percentage of cases correctly classified (PCCC) for traits days to heading (DTHD), days to maturity (DTMT) and Height and in terms of average Pearson’s correlation for trait grain yield (GY). (**A**) Prediction accuracy of MTDLMP and UDL models with the G×E term (I) and without (WI) for each trait with 1 layer; (**B**) prediction accuracy with different numbers of layers (1, 2 and 3) across environments with the MTDLMP model with the G×E term (I) and without the G×E term (WI); and (**C**) prediction accuracy obtained with different numbers of layers (1, 2 and 3) with the UDL model with the G×E term (I) and without the G×E term (WI).

The pattern of genomic-enabled prediction accuracy is similar to that found in **data set 1**. For GY with (I) and without (WI) G×E interaction, the best prediction was provided by the MTDLMP model, which outperformed the UDL model by 19.21% with interaction (I) and by 40.02% without interaction (WI) (Figure 5a). However, for trait GY with 2 layers, there were significant differences in favor of the MTDLMP model with the interaction term (I) (Figure D2_SA; Supplementary material, hdl:11529/10548140), while with 3 layers, no significant differences were observed between the MTDLMP and the UDL models for GY (Figure D2_SB; Supplementary material, hdl:11529/10548140). The PCCC ranged from 0.6071 to 0.7124 using 1 layer for the binary and ordinal traits (DTHD, DTMT and Height), and from 0.3301 to 0.5504 for trait GY in terms of APC (Figure 5a). Also, when comparing the prediction accuracy using different numbers of layers (1, 2, and 3) under the MTDLMP model (Figure 5b), we only found statistical differences in terms of prediction performance when using 1, 2 or 3 layers in both models with (I) and without (WI) interaction term in trait GY; the worst prediction was observed with 3 layers, and the best when using 1 layer. Also under the UDL model (Figure 5c), we only found statistical differences when using 1, 2 or 3 layers in trait GY with (I) and without (WI) the interaction term, and the best predictions were observed using 3 layers with the interaction term (I) and with 2 and 3 layers without the interaction term (WI).

### Data set 3

**Figure 6:**
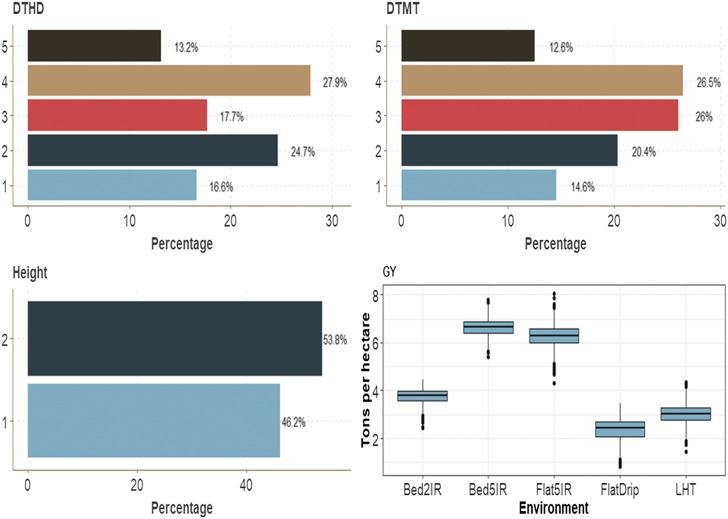
Percentage of each ordinal response for *data set 3* across environments for traits days to heading (DTHD), days to maturity (DTMT) and Height. Boxplot of trait grain yield (GY) for each environment.

This data set also had four traits; the trait Height (binary trait) had 46.2% and 53.8.2% of observations in categories 1 and 2, respectively, across environments. The distribution pattern across the individuals in the five categories of ordinal variables DTHD and DTMT was similar. The average GY was above 6 ton/ha in environments Bed5IR and Flat5IR, around 4 ton/ha in environment Bed2IR and less than 3 tons/ha in environments FlatDrip and LHT (Figure 6).

**Figure 7:**
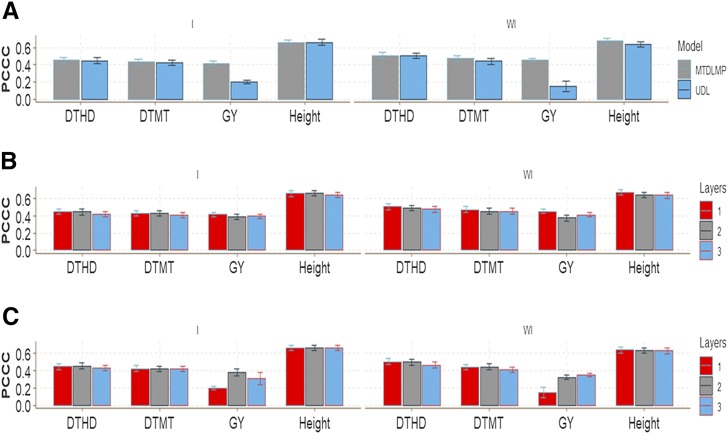
Prediction accuracy of *data set 3* in terms of percentage of cases correctly classified (PCCC) for traits days to heading (DTHD), days to maturity (DTMT) and Height and in terms of average Pearson’s correlation for trait grain yield (GY). (**A**) Prediction accuracy of MTDLMP and UDL models with the G×E term (I) and without (WI) for each trait with 1 layer; (**B**) prediction accuracy with different numbers of layers (1, 2 and 3) across environments with the MTDLMP model with the G×E term (I) and without the G×E term (WI); and (**C**) prediction accuracy obtained with different numbers of layers (1, 2 and 3) with the UDL model with the G×E term (I) and without the G×E term (WI).

The genomic-enabled prediction accuracy for GY with 1 and 2 layers under MTDLMP and UDL was significantly different with (I) and without (WI) the G×E interaction term; the best predictions were observed under the MTDLMP model, which outperformed the UDL model by 52.92% (with 1 layer), by 1.97% (with 2 layers) and by 22.21% (with 3 layers) with the interaction term (I), and by 67.27% (with 1 layer), by 13.34% (with 2 layers) and by 15.16% (with 3 layers) without the interaction term (WI) (Figure 7a; Figures D3_SA and D3_SB, Supplementary material, hdl:11529/10548140). The PCCC ranged from 0.4259 to 0.6746 using 1 layer for the binary and ordinal traits (DTHD, DTMT and Height), while it ranged from 0.1484 to 0.4535 for GY in terms of APC (Figure 7a). Also, when comparing the prediction accuracy using different numbers of layers (1, 2, and 3) under the MTDLMP model (Figure 7b), we found no statistical differences in terms of prediction performance between using 1, 2 or 3 layers in the four traits with (I) and without (WI) the G×E interaction term. Under the UDL model (Figure 7c) we only found statistical differences between using 1, 2 or 3 layers in trait GY with (I) and without the interaction term (WI).

### Data set 4

**Figure 8:**
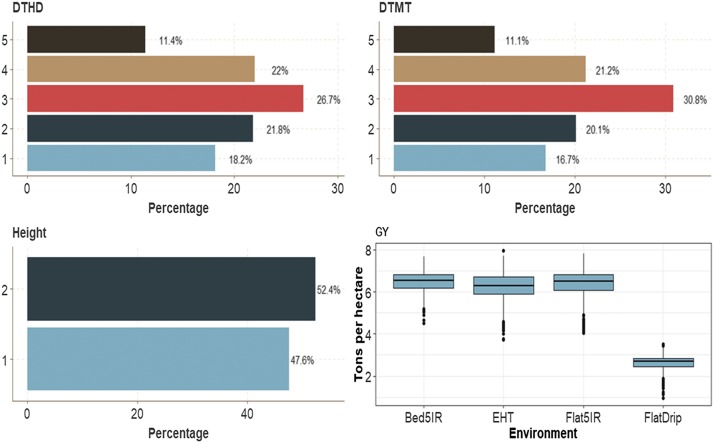
Percentage of each ordinal response for *data set 4* across environments for traits days to heading (DTHD), days to maturity (DTMT) and Height. Boxplot of trait grain yield (GY) for each environment.

In trait Height (binary trait), the first category across environments had 47.6% of cases, while category 2 had 52.4% of cases. As in the previous data sets, the distribution of individuals for both ordinal traits DTHD and DTMT in each category was similar. The average GY (continuous trait) was 6 ton/ha in three environments (Bed5IR, EHT and Flat5IR) and less than 3 ton/ha in environment FlatDrip (Figure 8).

**Figure 9:**
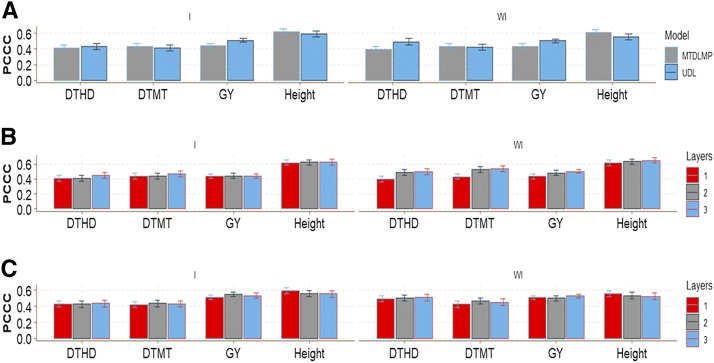
Prediction accuracy of *data set 4* in terms of percentage of cases correctly classified (PCCC) for traits days to heading (DTHD), days to maturity (DTMT) and Height and in terms of average Pearson’s correlation for grain yield (GY) trait. (**A**) Prediction accuracy of MTDLMP and UDL models with the G×E term (I) and without the G×E term (WI) for each trait with 1 layer; (**B**) prediction accuracy with different numbers of layers (1, 2 and 3) across environments with the MTDLMP model with the G×E term (I) and without the G×E term (WI); and (**C**) prediction accuracy obtained with different numbers of layers (1, 2 and 3) with the UDL model with the G×E term (I) and without the G×E term (WI).

In this data set, in terms of prediction performance using 1 layer when the G×E interaction was taken into account (I), there were only significant differences between the MTDLMP and UDL models in trait GY, and the UDL model outperformed the MTDLMP model by 13.51% (Figure 9a). However, when the G×E interaction was ignored (WI), statistical differences were found in two (DTHD and GY) out of four traits, with a better performance under the UDL model (Figure 9a). In terms of prediction accuracy, the UDL model outperformed the MTDLMP model by 6.24% and 14.39% for traits DTHD and GY, respectively. With 2 layers, we found statistical differences between the MTDLMP and UDL models in trait GY with the interaction term (I), while the UDL model was 18.81% better than the MTDLMP; without the interaction term, we only found differences in trait Height where the MTDLMP model outperformed the UDL model by 15.71% (Figure D4_SA; Supplementary material, hdl:11529/10548140). With 3 layers and the interaction term (I), we found statistical differences between the two models in trait GY, for the UDL model outperformed the MTDLMP model by 17.71%, (Figure D4_SA; Supplementary material, hdl:11529/10548140). Without the interaction term (WI) in two traits (DTMT and Height), we found statistical differences between the two models and in both traits: the MTDLMP model outperformed the UDL model by 15.93% in trait DTMT and by 19.18% in trait Height (Figure D4_SB; Supplementary material, hdl:11529/10548140). The PCCC using 1 layer for the binary and ordinal traits (DTHD, DTMT and Height) ranged from 0.3974 to 0.6197, while the APC for the GY trait ranged from 0.4346 to 0.5105 (Figure 9a). When comparing the prediction accuracy using different number of layers (1, 2, and 3) under the MTDLMP model (Figure 9b), no statistical differences in terms of prediction performance were found between using one, two or three layers with the G×E interaction term (I), but without the interaction term (WI) statistical differences were found between using 1, 2, and 3 layers in traits DTHD, DTMT and GY with lower predictions with 1 layer. However, under the UDL model (Figure 9c), there were no statistical differences between using one, two and three layers.

### Data set 5

**Figure 10:**
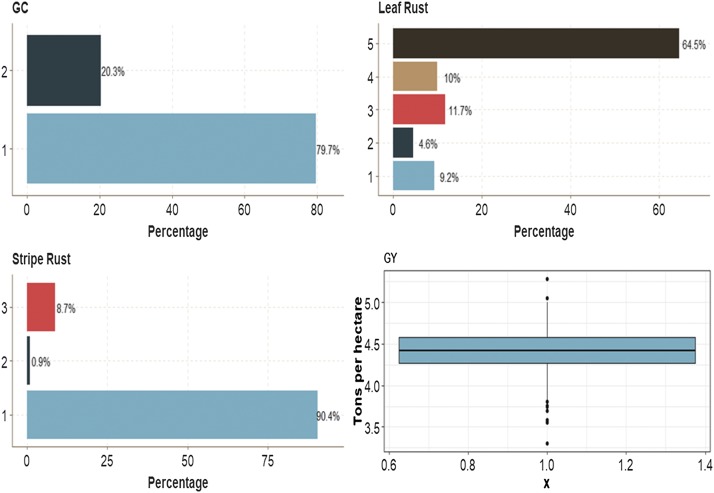
Percentage of each ordinal response for *data set 5* for traits grain color (GC), Leaf Rust and Stripe Rust. Boxplot of grain yield (GY).

This data set had four traits with mixed phenotypes. In binary trait GC, the first category had 79.7% of the cases, while category 2 had 20.3% of cases. For ordinal trait Leaf Rust, the first category had 9.2% of the cases, category 2 had 4.6% of the cases, category 3, 11.7% of the cases, and category 4, 10.0% of the cases. The most numerous category was category 5, with 64.5% of the cases. On the other hand, the first category of trait Stripe Rust had 90.4% of the cases, whereas category 2 and category 3 had 0.90% and 8.7% of individuals, respectively. The average of the continuous trait GY was around 4.4 ton/ha (Figure 10).

**Figure 11:**
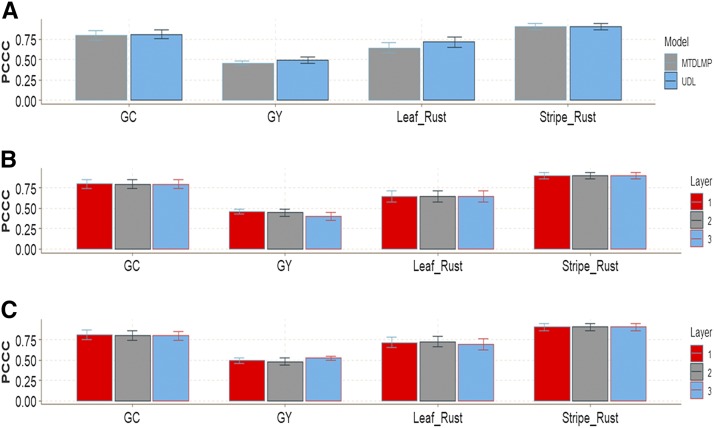
Prediction accuracy of *data set 5* in terms of percentage of cases correctly classified (PCCC) for traits grain color (GC), Leaf Rust and Stripe Rust and in terms of average Pearson’s correlation for trait grain yield (GY). (**A**) Prediction accuracy between the MTDLMP and UDL models with 1 layer; (**B**) prediction accuracy with different numbers of layers (1, 2 and 3) with the MTDLMP model; and (**C**) prediction accuracy obtained with different numbers of layers (1, 2 and 3) with the UDL.

In this data set, in terms of prediction performance using 1 and 2 layers, no significant differences were found in any trait between the MTDLMP and UDL models (Figure 11a; Figure D5_SA; Supplementary material, hdl:11529/10548140). With 3 layers, there were statistical differences between the two models only for trait GY, where the UDL model outperformed the MTDLMP model by 23.40%, (Figure D5_SB; Supplementary material, hdl:11529/10548140). The PCCC using 1 layer for the binary and ordinal traits (GC, Leaf Rust, Stripe Rust) ranged from 0.6442 to 0.9035, while the APC for the GY trait ranged from 0.4574 to 0.4920 (Figure 10a). When comparing the prediction accuracy using different numbers of layers (1, 2 and 3) under the MTDLMP model (Figure 11b) and the UDL model (Figure 11C), there were no statistical differences in terms of prediction performance between using one, two or three layers.

### Data set 6

**Figure 12:**
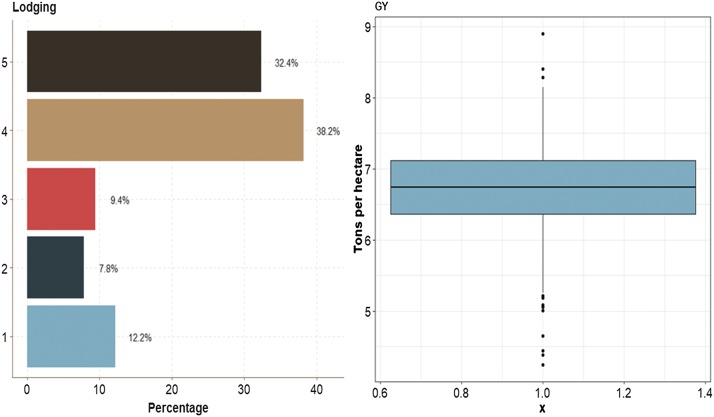
Percentage of the ordinal response (Lodging) for *data set 6* and boxplot of grain yield (GY).

This data set had two traits (one ordinal and one continuous). The ordinal trait (Lodging) had 12.2% of cases in category 1, 7.8% in category 2, 9.4% in category 3, 38.2% in category 3 and 32.4% in category 5. For continuous trait GY, the average was around 6.7 ton/ha (Figure 12).

**Figure 13:**
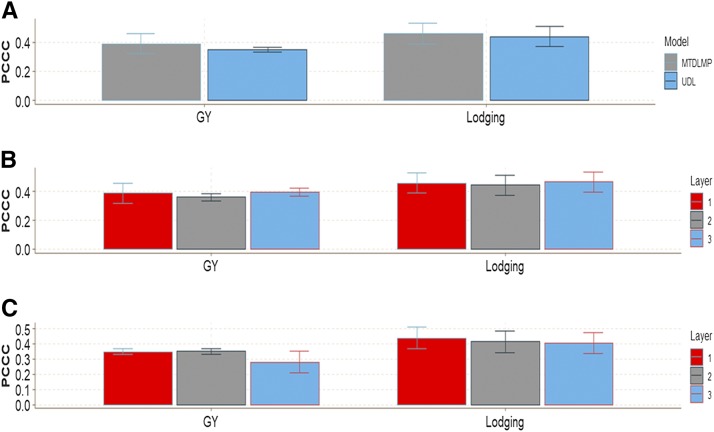
Prediction accuracy of *data set 6* in terms of percentage of cases correctly classified (PCCC) for trait Lodging and in terms of average Pearson’s correlation for grain yield GY. (**A**) Prediction accuracy between the MTDLMP and UDL models with 1 layer; (**B**) prediction accuracy with different numbers of layers (1, 2 and 3) with the MTDLMP model; and (**C**) prediction accuracy obtained with different numbers of layers (1, 2 and 3) with the UDL.

In terms of prediction performance, in **data set 6** when using 1 and 2 layers, there were no significant differences in any trait between the MTDLMP and UDL models (Figure 13a; Figure D6_SA Supplementary material, hdl:11529/10548140). With 3 layers, there were statistical differences between the two models only in GY, where the MTDLMP model outperformed the UDL model by 29.78% (Figure D6_SB, Supplementary material, hdl:11529/10548140). The PCCC using 1 layer for the ordinal trait (Lodging) ranged from 0.4392 to 0.4603, while the APC for the GY trait ranged from 0.3491 to 0.3896 (Figure 13a). Also, comparing the prediction accuracy using different numbers of layers (1, 2, and 3) under the MTDLMP (Figure 13b) and UDL (Figure 13C) models, there were no statistical differences in terms of prediction performance using 1, 2 or 3 layers.

### Data set 7

**Figure 14:**
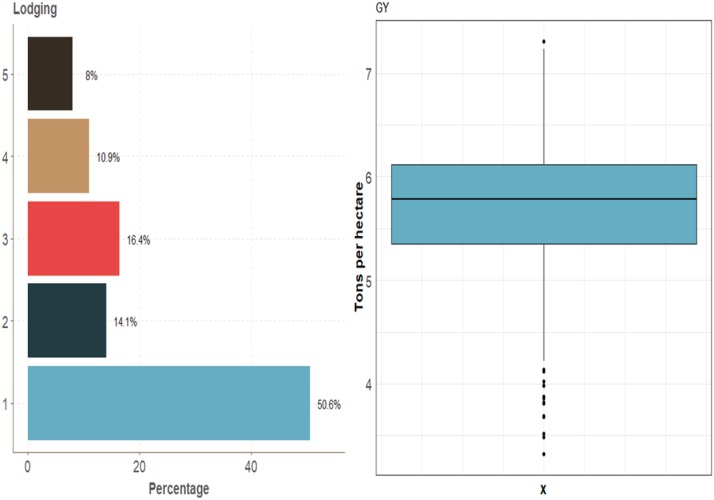
Percentage of each ordinal response for *data set 7* for traits grain color (GC), Leaf Rust and Stripe Rust and boxplot of grain yield (GY).

This real data set had two traits (one ordinal and one continuous). The ordinal trait (Lodging) had 50.6%, 14.1%, 16.4%, 10.9% and 8.0% of individuals in categories 1, 2, 3, 4 and 5, respectively. The continuous trait GY had an average of around 5.75 ton/ha (Figure 14).

**Figure 15:**
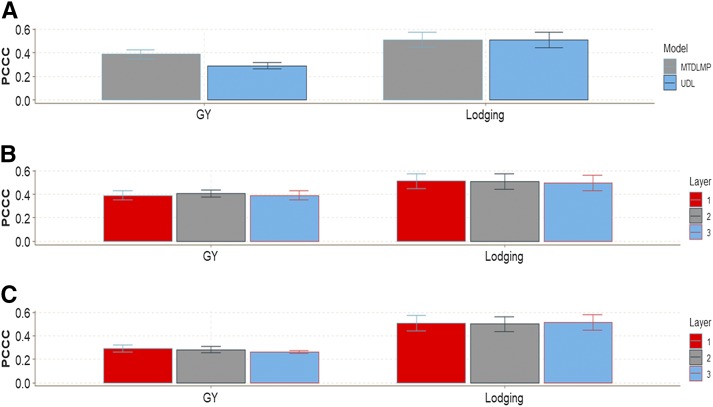
Prediction accuracy of *data set 7* in terms of percentage of cases correctly classified (PCCC) for trait Lodging and in terms of average Pearson’s correlation for grain yield (GY). (**A**) Prediction accuracy between the MTDLMP and UDL models with 1 layer; (**B**) prediction accuracy obtained with different numbers of layers (1, 2 and 3) with the MTDLMP model; and (**C**) prediction accuracy obtained with different numbers of layers (1, 2 and 3) with the UDL.

There were statistical differences in terms of genome-enabled prediction accuracy between models MTDLMP and UDL only for trait GY (Figure 15a; Figures D7_SA, and Figure D7_SB, Supplementary material, hdl:11529/10548140). Model MTDLMP outperformed model UDL by 25.14%, 30.46% and 32.83% when using 1, 2 and 3 layers, respectively. The PCCC using 1 layer for the ordinal trait (Lodging) ranged from 0.5082 to 0.5117, while the APC for the GY trait ranged from 0.2914 to 0.3893 (Figure 15a). When comparing the prediction accuracy using different numbers of layers (1, 2 and 3) under the MTDLMP (Figure 15b) and UDL (Figure 15c) models, no statistical differences were found in genomic-enabled prediction performance accuracy using 1, 2 and 3 layers.

## Discussion

In genomic prediction, various approaches have been developed for increasing prediction accuracy mainly of continuous traits (for example, all models under the Bayesian alphabet). However, few approaches have been developed for non-normal phenotypes and multivariate prediction of mixed phenotypes: binary, ordinal and continuous. In the context of mixed phenotypes, the most common approach used is to perform a separate univariate analysis for each trait; this ignores the correlations among multiple traits. For this reason, in this paper we propose using multiple-trait deep learning for the prediction of mixed phenotypes: binary, ordinal and continuous. We compare the prediction performance of the MTDLMP model with those of the UDL model using fivefold cross-validation in seven data sets using the PCCC (for binary and ordinal phenotypes) and Pearson’s correlation (for continuous phenotypes) as metrics for measuring the prediction performance. In four data sets, we discretized some continuous traits to make them ordinal and binary, while in the other three data sets, discretization was not necessary since these three data sets naturally contain binary, ordinal and continuous traits.

Our results using seven data sets showed that using multiple-trait deep learning is a practical approach for simultaneously predicting multiple traits with mixed phenotypes (binary, ordinal and continuous), given that the predictions obtained under the MTDLMP model are not low. The gain in terms of prediction performance of the MTDLMP over the UDL model was intermediate, given that when the G×E interaction term was taken into account, the MTDLMP was better than the UDL model in 4 out of the 7 data sets, while when the G×E interaction term was ignored, the MTDLMP was better in 5 out of the 7 data sets.

It is also important to point out that the observed gain in terms of prediction performance of the MTDLMP over the UDL model was observed only in the continuous trait GY, while in the remaining traits no statistical differences were observed between the two deep learning models. Part of these results can be attributed in part to the fact that the phenotypic correlations between traits are not high since for data set 1 the minimum, average and maximum values were 0.103, 0.237 and 0.794, respectively, for data set 2 the minimum, average, and maximum values were -0.044, 0.166 and 0.782, respectively, for data set 3 the minimum, average, and maximum values were -0.219, 0.058 and 0.719, respectively, for data set 4 the minimum, average, and maximum values were -0.079, 0.179 and 0.803, respectively, for data set 5 the minimum, average, and maximum values were -0.245, -0.041 and 0.051, and for data set 6 and 7 the correlations between the two traits under study were -0.517 and -0.4056, respectively. Also, two of the weakness of our study is that the number of markers used in the 7 data sets is substantially low which may be affecting the prediction accuracy. For this reason, we are aware that more empirical evaluations are needed to have a better picture of the predictive power of the MTDLMP model. We also found that, in general, in these data sets increasing the number of hidden layers did not help to significantly increase the prediction accuracy, since in most situations that we evaluated, the best predictions were obtained with only one hidden layer.

To successfully implement the MTDLMP, the following issues need to be taken into account. The continuous traits need to be standardized (subtracting the mean and dividing by the standard deviation) in each training set when the response variable is not close to mean zero and the variance is equal to 1. Since it is necessary to specify different activation functions for continuous, binary and ordinal data, we used the ReLU activation function for continuous traits and the sigmoid and softmax activation functions for binary and ordinal traits. Different metrics must be used to measure the prediction performance for continuous and ordinal (and binary) traits, *i.e.*, Pearson’s correlation for continuous traits and percentage of cases correctly classified (PCCC) for ordinal and binary traits. The process for choosing the optimal (or near optimal) hyperparameters must be tuned in order to increase the chances of prediction.

It is not possible to do a formal comparison of the results of this study with those of Montesinos-López *et al.* (2018a, b). However, it should be noted that the prediction accuracies of the DL method with genotype × environment interaction for single traits such as grain yield, plant height, days to heading and days to maturity obtained by Montesinos-López *et al.* (2018a) are higher than the prediction accuracies found in this study. The prediction accuracies reported by Montesinos-López *et al.* (2018b) for the multiple-trait DL (MTDL) are similar to those reported in this study for ordinal data. Furthermore, the results where the MTDL without including genotype × environments is slightly superior in prediction accuracy to the Bayesian multiple-trait multiple-environment and vice versa (when the genotype × environments is ignored) agree with the results obtained in this study.

It should be noted that the proposed method for the simultaneous prediction of mixed phenotypes (binary, ordinal and continuous) under the deep learning (a type of machine learning method) framework is novel, since nowadays in plant breeding there are no statistical models available that are able to simultaneously predict mixed phenotypes, given that multiple-trait (multivariate) models have only been developed for continuous traits. It is important to point out that multivariate models for mixed response variables (traits) are available in the statistical literature under classic (maximum likelihood) and Bayesian methods. However, these available models are not appropriate for dealing with large data sets nor with the problem of large p and small n in genomic-enabled prediction, since those Bayesian models were not built with an analytical Gibbs sampler, due to the complexity of the likelihood function, which is a mixture of normal and other types of distributions.

Finally, this application of deep learning for the simultaneous prediction of mixed phenotypes (binary, ordinal and continuous) is important due to the fact that there is a lack of multivariate models for simultaneously predicting mixed phenotypes (binary, ordinal and continuous) in plant breeding and because we found that this model can be implemented using the open-source R statistical software with the keras package. Also, this package is easy to implement and does not require that its users have a strong computational and mathematical background. It is efficient in terms of the computing resources required and allows the implementation of other types of DL architectures, such as convolutional networks, recurrent networks, etc., that can help improve prediction accuracy in some circumstances.

## Conclusions

In this paper we propose applying deep learning for simultaneously predicting multiple traits with mixed phenotypes (binary, ordinal and continuous). This application is novel in GS since, to the best of our knowledge, nowadays there are no multiple-trait models available for the simultaneous prediction of mixed phenotypes. When comparing the prediction performance of the MTDLMP model with that of the UDL model, gains in terms of prediction accuracy were only obtained in trait grain yield, and no differences were detected in binary and ordinal traits. In general, the deep learning model for simultaneously predicting mixed phenotypes is an attractive alternative for breeders due to the lack of models for the simultaneous prediction of mixed phenotypes; the existence of friendly open-source software for its implementation is also an important advantage. For this reason, we believe deep learning models should be included in the toolkit of scientific breeders, since there is empirical evidence that there is no universally best prediction model for genomic-enabled prediction.
